# Exogenous glutathione improves high root-zone temperature tolerance by modulating photosynthesis, antioxidant and osmolytes systems in cucumber seedlings

**DOI:** 10.1038/srep35424

**Published:** 2016-10-18

**Authors:** Xiaotao Ding, Yuping Jiang, Lizhong He, Qiang Zhou, Jizhu Yu, Dafeng Hui, Danfeng Huang

**Affiliations:** 1 School of Agriculture and Biology, Shanghai Jiaotong University, Shanghai 202400, China; 2 Shanghai Key Lab of Protected Horticultural Technology, Horticultural Research Institute, Shanghai Academy of Agricultural Sciences, Shanghai 201106, China; 3 Department of Biological Sciences, Tennessee State University, Nashville, Tennessee 37209, United States of America

## Abstract

To investigate the physiological responses of plants to high root-zone temperature (HT, 35 °C) stress mitigated by exogenous glutathione (GSH), cucumber (*Cucumis sativus* L.) seedlings were exposed to HT with or without GSH treatment for 4 days and following with 4 days of recovery. Plant physiological variables, growth, and gene expression related to antioxidant enzymes and Calvin cycle were quantified. The results showed that HT significantly decreased GSH content, the ratio of reduced to oxidized glutathione (GSH/GSSG), chlorophyll content, photosynthesis and related gene expression, shoot height, stem diameter, as well as dry weight. The exogenous GSH treatment clearly lessened the HT stress by increasing the above variables. Meanwhile, HT significantly increased soluble protein content, proline and malondialdehyde (MDA) content as well as O_2_•^−^ production rate, the gene expression and activities of antioxidant enzymes. The GSH treatment remarkably improved soluble protein content, proline content, antioxidant enzymes activities, and antioxidant enzymes related gene expression, and reduced the MDA content and O_2_•^−^ production rate compared to no GSH treatment in the HT condition. Our results suggest that exogenous GSH enhances cucumber seedling tolerance of HT stress by modulating the photosynthesis, antioxidant and osmolytes systems to improve physiological adaptation.

Global warming is generally predicted to have a negative effect on plant growth and productivity due to the damaging effect of high temperature on plant development^1^,^2^. Under heat stress conditions, plants are subject to physical changes in their environments^3^ as well as reduction in performance of plant cell functions, including enzyme activity, membrane fluidity, protein complexes formation, chlorophyll synthesis, photosynthesis, respiration, and redox state^4^,^5^. Considering that global temperature continues to increase and there is an urgent need for the development of adaptation strategies to maintain agricultural yields, a better understanding of the influences of the high temperature on plant physiology and growth is required.

Cucumber (*Cucumis sativus* L.) is a thermophilic species which grows well in warmer condition, however it is intolerant to high temperature, especially in its root zone^6^. In order to fulfill the highly increasing demand of vegetables such as cucumber, greenhouse vegetable production systems have been rapidly developed in recent decades in China^7^. The greenhouse temperature can easily reach and exceed to high and intolerable level (above 38 °C) during the summer^8^. Opening the greenhouse windows is the common approach for reduction of the air temperature but not soil temperature, especially for soil which is covered with black films. Temperature in the root-zone is often above 30 °C, or even higher than 35 °C in hot seasons. High root-zone temperature (HT) may negatively affect plant physiology, morphology and growth^9^,^10^. But a comprehensive investigation of the physiological basis underlying cucumber plant responses to the HT stress is still lacking.

Photosynthesis is an important biological process that is highly sensitive to high temperature stress and is often inhibited before other cell functions are impaired^11^. The main reason might be that high temperature seriously suppresses the activities of ribulose-1,5-bisphosphate carboxylase/oxygenase (Rubisco) large subunit (RBCL), Rubisco small subunit (RBCS) and other related enzymes^12^, and leads to impairment of chlorophyll biosynthesis^13^. It has been reported that biotic and abiotic stress often induces the overproduction of reactive oxygen species (ROS) such as the superoxide radical (O_2_•^−^) and hydrogen peroxide (H_2_O_2_)^14^. Accumulation of ROS can damage lipids, proteins, nucleic acids, and carbohydrates, leading to destruction of plant cells^15^. ROS are scavenged by plant antioxidant defense systems which include non-enzymatic antioxidants (such as ascorbic acid, AsA; glutathione, GSH) and antioxidant enzymes (such as superoxide dismutase, SOD; catalase, CAT; ascorbate peroxidase, APX; glutathione reductase, GR; guaiacol peroxidase, G-POD)^16^,^17^. Proline accumulation has been reported during various stress conditions, including drought^18^ and heat^19^. Proline is important in cellular homeostasis (cell proliferation or cell death) and can act as a signaling molecule to modulate mitochondrial functions and trigger specific genes expression, which is essential for plant recovery from stresses^20^.

GSH and associated redox status have been reported to play an important role in cellular signaling pathways involved in many physiologic processes in plants under both optimal and stress conditions^21^. GSH is able to provide stress protection in several ways^22^,^23^. Reduced GSH acts as an antioxidant and is directly involved in reducing most ROS^24^. The ratio of reduced to oxidized glutathione (GSH/GSSG) redox couple is an indicator of the cellular redox status^5^,^25^. Some studies indicated that maintaining a high level of GSH or GSH/GSSG is important for the response to different stresses, such as drought tolerance in mung bean^26^, low temperature tolerance in loquat^27^, and isoproturon toxicity in wheat^28^. However, the role of GSH or GSH/GSSG levels in response to HT remains unknown.

In this study, a glasshouse experiment was conducted to test the HT and exogenous GSH effects on cucumber plant physiology and growth. We hypothesized that applying exogenous GSH to roots may increase heat tolerance by triggering the enzyme activities and gene expression level involved in photosynthesis and stress responses, as well as cellular redox homeostasis maintaining in plants. The specific objectives of this study were: (1) to investigate the effects of the HT on plant physiological variables; (2) to test whether exogenous GSH application can effectively enhance plant tolerance; and (3) to illustrate the potential adaption mechanism in HT stress. The information generated in this study could improve our understanding of HT tolerance of cucumber plants and would be useful for greenhouse and protected vegetation production system management.

## Results

### Effects of HT and exogenous GSH on plant growth

**Figure 1:**
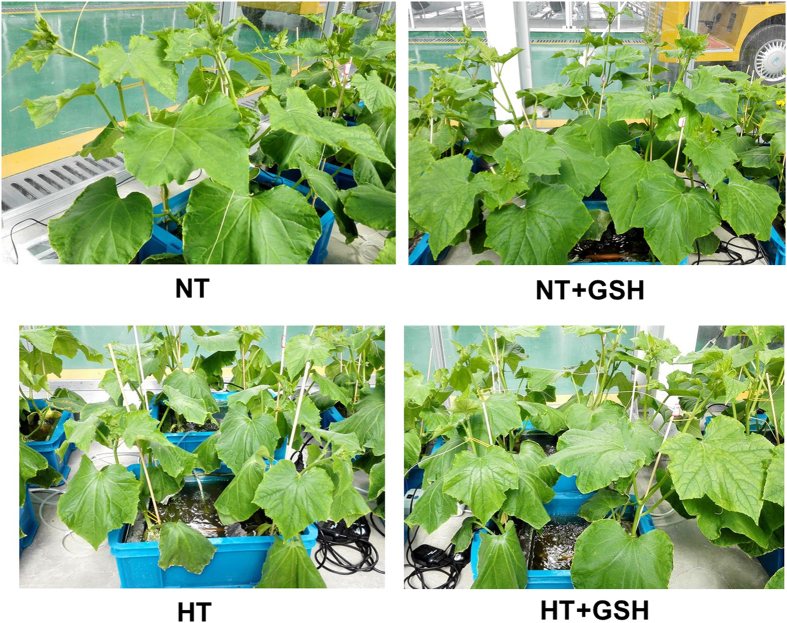
Photographs of cucumber seedlings under 4 treatments, 4 days after the high root-zone temperature treatment, demonstrating effects of exogenous GSH on the response to high root-zone temperature stress.

Compared to normal temperature treatment (NT), plant height, stem diameter, shoot fresh weight, and shoot dry weight of the HT treatment were significantly reduced by 14.8%, 8.1%, 38.1% and 32.6%, respectively, in the HT stress and by 24.0%, 12.6%, 33.5%, 37.7%, respectively, in the recovery period (Table 1; Fig. 1). High root-zone temperature plus exogenous GSH treatment (HT+GSH) significantly increased plant height, stem diameter, shoot fresh weight, and dry weight by 23.2%, 7.3%, 56.8% and 20.7% in the HT stress and by 29.2%, 7.7%, 32.0% and 25.7% in the recovery period, compared to the HT only treatment. Shoot water content was significantly decreased by 1.0% in the HT treatment compared to the NT treatment, however, there was no significant difference between them during the recovery period. HT+GSH treatment significantly increased the shoot water content by 2.7% in contrast with the HT treatment but showed no difference in the recovery period. There were no significant differences for any of the variables measured between the NT treatment and normal temperature plus exogenous GSH treatment (NT+GSH) during and after the HT stress. Shoot dry weight significantly decreased and shoot water content significantly increased for the HT+GSH treatment under the HT stress, and stem diameter, shoot fresh weight, and shoot dry weight were clearly decreased in the recovery period compared to the NT treatment.

### Effects of HT and exogenous GSH on photosynthetic tolerance

**Figure 2:**
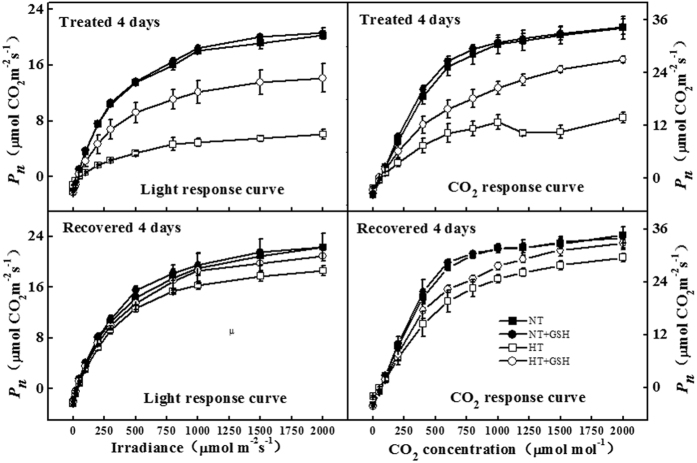
Changes of leaf photosynthetic rate (*P*_*n*_) to light response curve and CO_2_ response curve for different treatments 4 days after the high root-zone temperature treatment, and 4 days recovery. Data are means of three biological replications.

In the present study, changes in the light and CO_2_ response curves of leaf photosynthetic rate (*P*_*n*_) for different treatments were studied during and after the HT treatment (Fig. 2). Compared to the NT treatment, the light response curves showed that cucumbers experienced the strongest stress under the HT treatment, with *P*_*n*_ of only 6 μmol CO_2_ m^−2^s^−1^ under 2000 μmol m^−2^s^−1^ irradiance. The HT+GSH treatment enhanced *P*_*n*_ by a factor of 2.3 compared to HT. We derived the maximum leaf photosynthetic rate (*P*_*max*_), quantum yield (*α*), and the leaf photosynthetic rate under no irradiance (*P*_*0*_) from the light response curves using the rectangular hyperbolic equation^29^. Compared to the NT treatment, the HT treatment significantly decreased *P*_*0*_ (from 2.415 (NT) to 1.033 μmol CO_2_ m^−2^s^−1^ (HT)), *P*_*max*_ (from 26.61 (NT) to 8.58 μmol CO_2_ m^−2^s^−1^ (HT)) and *α* (from 0.0794 (NT) to 0.0194 (HT)) (Table 2). HT+GSH significantly increased *P*_*0*_ (2.174 μmol CO_2_ m^−2^s^−1^), *P*_*max*_ (19.20 μmol CO_2_ m^−2^s^−1^) and *α* (0.0556) compared to the HT treatment.

The CO_2_ response curve under the HT treatment showed the lowest *P*_*n*_ value among the treatments. The HT+GSH treatment had higher *P*_*n*_ than HT across CO_2_ levels (Fig. 2). In the HT treatment, we found that the maximum velocity of RuBP carboxylation by Rubisco (*V*_cmax_) and the maximum potential rate of electron transport contributing to RuBP regeneration (*J*_max_) also dropped significantly compared to the NT treatment (from 47.30 (NT) to 20.42 μmol m^−2^s^−1^ (HT) and from 126.63 (NT) to 45.51 μmol m^−2^s^−1^ (HT), respectively). Similar to the light responses, the HT+GSH treatment increased the values to 40.73 μmol m^−2^s^−1^ for *V*_cmax_ and 97.81 μmol m^−2^s^−1^ for *J*_*max*_ (Table 2).

In the recovery period, cucumber *P*_*n*_ was enhanced based on the light and CO_2_ response curves, and *P*_*n*_ in the HT+GSH treatment was increased more than in the HT treatment during the HT stress. As a result, there were no differences in *P*_*0*_ and *α* under all treatments, and *P*_*max*_, *V*_*cmax*_ and *J*_*max*_ were slightly decreased under the HT treatment compared to the NT treatment. The HT+GSH treatment still slightly increased *P*_*max*_, *V*_*cmax*_ and *J*_max_ compared to the HT treatment. There were no significant differences in *P*_*0*_, *P*_*max*_, *α*, *V*_*cmax*_, and *J*_*max*_ between the NT and NT+GSH in both treatment and recovery periods (Fig. 2 and Table 2).

### Effects of HT and exogenous GSH on chlorophyll, carotenoid, soluble protein, and proline content

Compared to the NT treatment, the HT treatment decreased leaf chlorophyll and carotenoid content significantly by 5.9% and 4.8%, respectively (Table 3). Soluble protein and proline content were increased by 15.4% and 24.0% respectively under the HT treatment. The HT+GSH treatment increased chlorophyll, soluble protein, and especially proline content, which was stimulated by 51.8% compared to the HT treatment. There were no differences in soluble protein or proline content between the NT+GSH and NT treatments. Chlorophyll and carotenoid contents were enhanced significantly under the NT+GSH treatment relative to the NT treatment.

### Effects of HT and exogenous GSH on antioxidant enzymes activities, MDA content, O_2_•^−^ production rate, and GSH redox homeostasis

**Figure 3:**
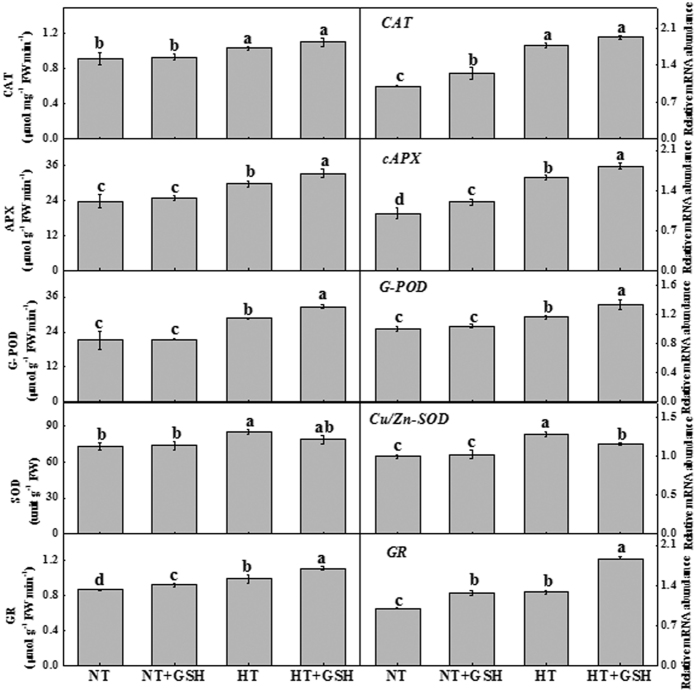
Effects of GSH application on catalase (CAT), ascorbate peroxidase (APX), superoxide dismutase (SOD), guaiacol peroxidase (G-POD) and glutathione reductase (GR) activities and their transcription levels changes in cucumber plants 4 days after the high root-zone temperature treatment. Data represent mean ± SD (n = 3). Different letters indicate significant differences at P < 0.05 based on the Least Significant Difference test.

The activities of CAT, APX, G-POD, SOD and GR were stimulated by 13.0%, 25.2%, 35.4%, 16.6% and 14.4%, respectively, under the HT treatment, and by 20.3%, 40.1%, 54.2%, 7.8% and 28.2%, respectively, under the HT+GSH treatment compared to the NT treatment (Fig. 3). The HT+GSH treatment significantly increased the activities of APX, G-POD, and GR, but had no obvious effect on CAT and SOD activity relative to the HT treatment. Although the antioxidant enzyme activities under the NT+GSH treatment were slightly increased, there was no significant difference between the NT+GSH and NT treatments for of the variables except GR.

**Figure 4:**
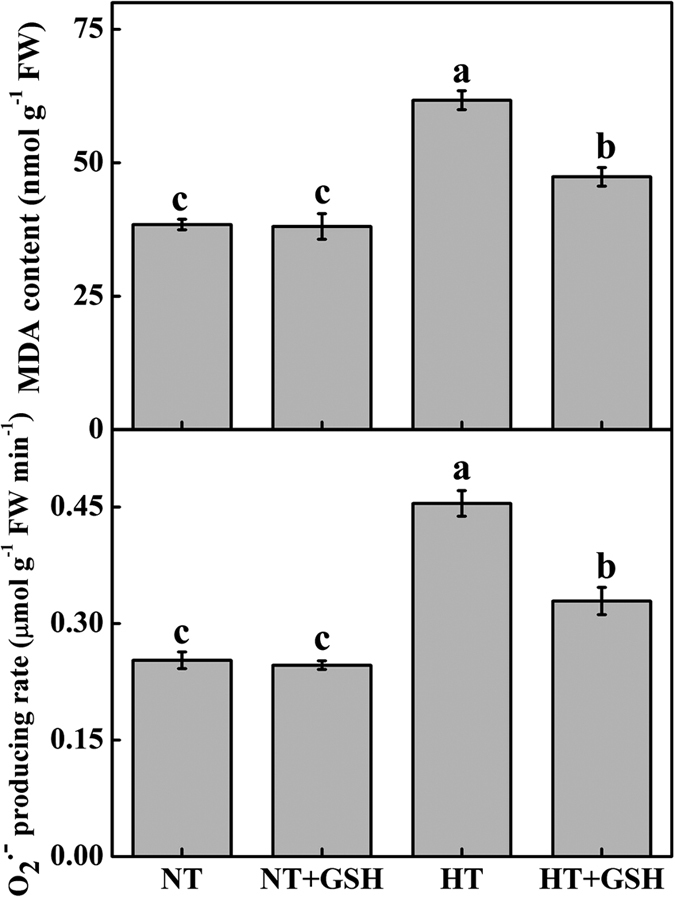
Effects of GSH application on MDA content and O_2_•^−^ production rate of cucumber 4 days after the high root-zone temperature treatment. Data represent the mean ± SD (n = 3). Different letters indicate significant differences at P < 0.05 based on the Least Significant Difference test.

Malondialdehyde (MDA) and O_2_•^−^production were significantly increased by 60.6% and 79.9%, respectively, under the HT treatment, and by 23.2% and 30.2%, respectively, under the HT+GSH treatment, compared to the NT treatment (Fig. 4). There was no obvious difference in MDA content and O_2_•^−^ production between the NT and NT+GSH treatments.

**Figure 5:**
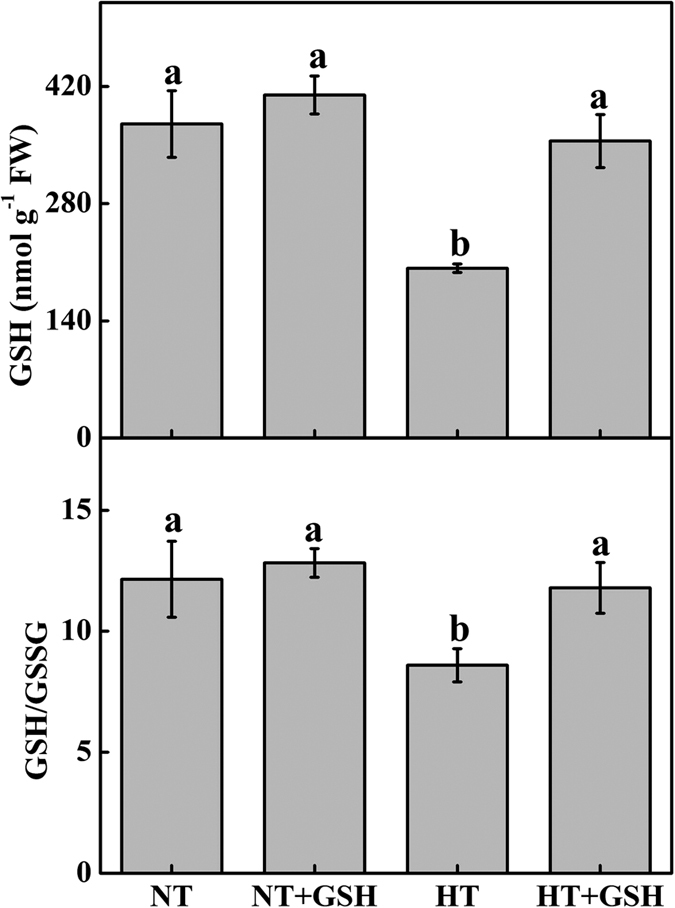
Effects of GSH application on GSH content and GSH/GSSG of cucumber 4 days after the high root-zone temperature treatment. Data represent the mean ± SD (n = 3). Different letters indicate significant differences at P < 0.05 based on the Least Significant Difference test.

The HT treatment remarkably decreased the level of GSH content and GSH/GSSG ratio in cucumber leaves compared to the NT treatment (Fig. 5). The HT+GSH treatment significantly enhanced the GSH content relative to the HT treatment, but had no significant difference compared to the NT or NT+GSH treatment.

### Effects of HT and exogenous GSH on gene expression related to photosynthesis and antioxidant enzymes

**Figure 6:**
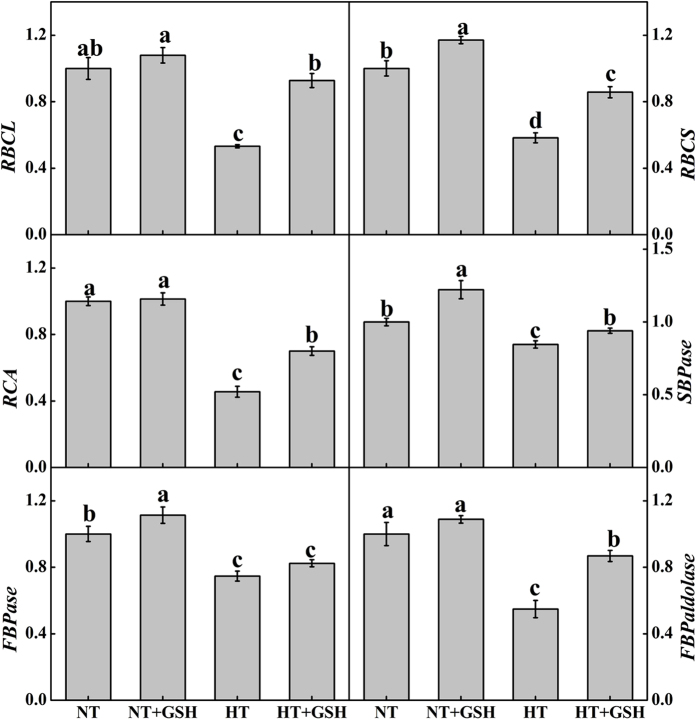
GSH application induced up-regulation of transcript levels for *RCBL*, *RCBS*, *RCA*, *SBPase*, *FBPase* and *FBPaldolase* 4 days after the high root-zone temperature treatment. Data represent the mean ± SD (n = 3). Different letters indicate significant differences at P < 0.05 based on the Least Significant Difference test.

The expression level of all the Calvin cycle enzyme genes investigated here were significantly up-regulated during the HT stress under the HT+GSH treatment compared to the HT treatment (except for fructose-1,6-bisphosphatase (*FBPase*) for which the increase was not significant) (Fig. 6), but all of the genes, to some extent, were down-regulated compared to the NT treatment. The gene expressions of *RBCS*, sedoheptulose-1,7-bisphosphatase (*SBPase*), and *FBPase* were significantly increased under the NT+GSH treatment compared to the NT treatment.

The transcription levels of antioxidant enzyme genes such as *CAT, cAPX* (cytoplasm ascorbate peroxidase)*, G-POD, Cu*/*Zn-SOD* and *GR* were significantly increased under the HT treatment relative to the NT treatment (Fig. 3). HT+GSH considerably enhanced the expressions of *CAT, cAPX, G-POD,* and *GR*, with *GR* being induced the most compared to the HT treatment. Compared to the NT treatment, the expressions of *CAT, cAPX,* and *GR* were significantly increased under the NT+GSH treatment.

## Discussion

As predicted, we found that the HT treatment significantly reduced plant height, stem diameter, shoot fresh weight, shoot dry weight, and shoot water content of cucumber, similar to some previous studies (e.g., Wahid *et al*.^30^). However, these reductions in plant growth were significantly alleviated by the addition of exogenous GSH (Table 1). The production of GSH under the HT stress is likely a critical mechanism for stress tolerance^5^,^31^ and has positive effects on plant growth and yield^32^.

Photosynthesis of cucumber plants was depressed under the HT treatment based on the light and CO_2_ response curves, which could be due to the reduction of chlorophyll content and carotenoid content at high temperatures that impaired the biosynthesis of photosynthetic pigments^33^. The application of exogenous GSH relieved the negative effects of the HT treatment on photosynthesis, not only during the HT treatment but also in the recovery period (Fig. 2). The HT+GSH treatment significantly increased chlorophyll and carotenoid content consistent with the results of previous studies^27^,^34^. The reduction of photosynthetic capacity was often accompanied by decreases in *P*_*max*_ and *α*^29^, *V*_cmax_ and *J*_max_^35^. Inactivation or loss of Rubisco would reduce *V*_cmax_ while a reduction in *J*_max_ is associated with the diminution of key regulatory enzymes such as SBPase and FBPase in the Calvin cycle^36^,^37^. Our results showed that *P*_*max*_, *α*, *V*_cmax_ and *J*_max_ were significantly decreased in the HT treatment and increased again in the HT+GSH treatment, which suggested that the enzymes involved in RuBP carboxylation by Rubisco and RuBP regeneration limit photosynthesis under the HT stress. A similar result was found by Li *et al*.^12^ who revealed that the activity of enzymes involved in CO_2_ assimilation is higher at cucumber optimal growth temperature. In addition, expressions of Calvin cycle enzyme genes such as *RBCL* and *RBCS* were significantly enhanced under the HT+GSH treatment, indicating that up-regulation of Calvin cycle enzymes at the translational level could positively influence CO_2_ assimilation^38^. This is consistent with previous studies that found transcription factors play an important role in biotic and abiotic stress responses^30^,^39^. Jiang *et al*.^24^ also reported positive relationships between the expressions of Calvin cycle enzyme genes and the GSH/GSSG ratio, and an important role of reducing redox state on the stability of Calvin cycle enzymes.

Our results showed that the HT treatment increased MDA content and O_2_•^−^ production rate in cucumber leaves, which was significantly decreased in the HT+GSH plants (Fig. 4). This is in accordance with previous findings that an increase in MDA level due to lipid peroxidation is one of the most common markers of cellular oxidative damage under the heat stress^40^. A higher GSH level reduces oxidative stress induced by heat stress^32^. Plants typically accumulate ROS under stress conditions. Tolerance to high temperature stress in plants is often associated with an increase in antioxidant enzyme activities^41^. In this study, we found that exogenous GSH increased the activities of CAT, APX, G-POD and GR under the HT treatment, which is consistent with the study of Nahar *et al*.^32^ who reported that oxidative damage effects are reduced by the GSH treatment during heat stress. Furthermore, transcription regulation of the antioxidant response (*cAPX, G-POD* and *GR*) was significantly increased under the HT+GSH treatment, which suggests that antioxidant adjustment in supra-optimal temperature is very important for plant adaptation to heat stress^5^. Huang *et al*.^19^ found that glucose application increases the transcriptions of some antioxidant enzymes under heat stress. The up-regulation of these genes contributed to an enhanced adaptation to high temperature stress in cucumber roots. Similarly, an exogenous GSH-induced improvement of activities of antioxidant enzymes reduces oxidative stress under various abiotic stress conditions^26^,^27^,^28^. Therefore, exogenous GSH regulates the accumulation of ROS and decreases membrane lipid peroxidation in the heat-stressed cucumbers through inducing antioxidant enzymes.

It is well established that proline accumulates in many plant species in response to environmental stress^20^. In this study, we found that proline was increased significantly under the HT treatment and even more when imposed in the HT+GSH treatment. These results indicated that the more accumulation of proline, the more stress tolerant for the plants. The reason could be that proline is an ROS scavenger and a molecular chaperone stabilizing the structure of proteins, thus helps protect cells from damage caused by the stress^20^. Similar results are reported in other plants such as mung bean^32^.

The accumulation of soluble protein, especially for the heat shock proteins during heat stress, contributes to stress tolerance in plants^30^. Our study demonstrated that the HT+GSH treatment significantly increased the soluble protein content of leaves under the HT stress, which is in agreement with a previous study that found protein protection plays an important role in maintaining high heat resistance in cucumber^5^.

The amelioration of exogenous GSH on the growth of cucumber under the HT treatment was effective (Fig. 1). The level of GSH has been shown to correlate with the adaptation of plants to extremes of temperature stresses^15^ and plants with a higher GSH/GSSG ratio possess higher stress-tolerance characteristics^26^. In this study, the HT+GSH treatment increased GSH accumulation and GSH/GSSG ratio compared to the HT treatment (Fig. 5). GR often plays an important role in the protection of plants from temperature stresses by preventing the oxidation of enzymes and membranes^42^. Indeed, higher GR activity was found with the application of exogenous GSH (Fig. 3), which may contribute to the high level of GSH and high GSH/GSSG ratio in the HT+GSH treatment compared to the HT treatment. Exogenous GSH application with drought^26^ and isoproturon toxicity^28^ also reduces these stresses by increasing GSH content and GSH/GSSG ratio.

**Figure 7:**
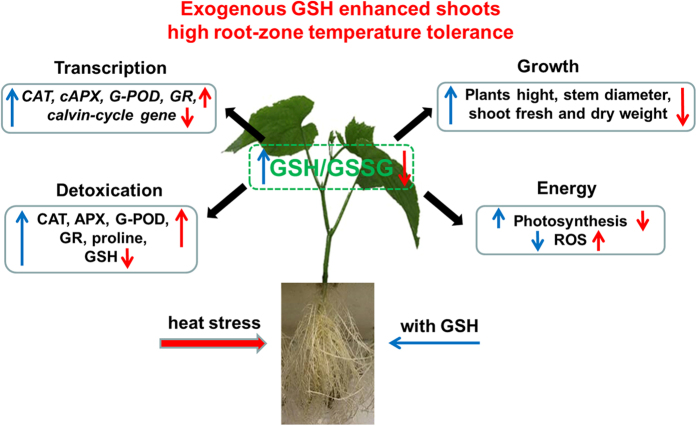
A framework of the effects of exogenous GSH on the responses of high root-zone temperature stress to physiological variables, antioxidant enzymes, and gene expressions of cucumber seedlings. Red arrow indicates the effects of HT treatment compared to the NT treatment. Blue arrow indicates the effects of HT+GSH treatment compared to the HT treatment. Up arrow means increased effect and down arrow means decreased effect.

In conclusion, we found that the stress of HT on cucumber seedlings was mostly relieved by the exogenous supplementation of GSH. Several mechanisms might be involved, including: (1) Maintaining high leaf water content, chlorophyll content, carotenoid content, and high activity of the Calvin cycle with the GSH application improved cucumber plant tolerance to the HT treatment, and led to high photosynthesis. GSH maintained osmotic balance by regulating soluble protein content and proline content thus improved the water status of plant leaves to resist the stress; (2) Exogenous GSH effectively eliminated HT-induced oxidative damage not only by increasing activities of antioxidant enzymes but also by improving transcription regulation of the antioxidant responses; and (3) Increases in GSH, enhanced GSH/GSSG ratio, or both, might be an obligatory event in the modulation of redox potential necessary for plants to adapt to the HT stress (Fig. 7). Such exogenous GSH to enhance HT tolerance mechanisms could be further exploited to improve our understanding of stress tolerance and the agricultural production especially in hot seasons.

## Materials and methods

### Plant material and treatments

The cucumber variety used in this study was Chunqiuwang NO. 2 selected by the Horticultural Research Institute of Shanghai Academy of Agricultural Sciences, China. Cucumber seeds were sown in Grodan blocks (10 cm × 10 cm × 6.5 cm) on February 12, 2015 in a well heated glasshouse. The seedlings were watered with half-strength Enshi nutrient solution^43^. The temperature in the glasshouse was maintained at 25 °C during the day and 18 °C at night. Plants grew under the glasshouse natural light. When the second true leaf was fully expanded, 4 seedlings were transplanted into each plastic container (27 cm × 40 cm × 12 cm). Nutrient solution was maintained full in the container, and a ventilation pump ensured the solution had enough oxygen. Meanwhile, a heater stick was fixed at the bottom of the container to control the solution temperature. The HT treatments were started when the fourth true leaf of plants was fully expanded. The treatments lasted 4 days, and measurements continued for 4 more days to capture the recovery period. The biggest leaves of each treatment were harvested after 4 days HT treatment, and the samples were frozen immediately in liquid nitrogen and stored at −80 °C for further analysis.

The experiment used a completely randomized design with three replications (plastic containers) for each treatment. Four treatments were included in this experiment: (1) normal temperature (NT): plants shoots grew in the normal glasshouse temperature, no heating for roots zone (about 22 °C with a range of 20–25 °C); (2) normal temperature plus exogenous GSH treatment (NT+GSH): added 0.25 mM GSH in nutrient solution one day before the treatments and maintained at 0.25 mM GSH in the nutrient solution during the treatment period; (3) high root-zone temperature treatment (HT): root-zone was heated to 35 ± 1 °C by heater stick; and (4) high root-zone temperature plus exogenous GSH treatment (HT+GSH): root-zone temperature was raised to 35 ± 1 °C and 0.25 mM GSH in nutrient solution was maintained.

### Growth and shoot water content measurements

Plant height, stem diameter, shoot fresh weight were measured after 4 days of treatments and 4 days of recovery. Meanwhile, shoots of each treatment were harvested for the measurement of fresh weight, and then placed in the oven at 80 °C for 3 days to measure the dry mass. Shoot water content was calculated as (plant fresh weight - dry weight)/plant fresh weight. Five replicates were performed for each treatment.

### Measurements of light response curve and CO_2_ response curve

Light and CO_2_ response curves were measured using a LI-6400 Portable Photosynthesis System (Li-Cor Inc., Lincoln, NE, USA) on the middle fully developed leaves of cucumber seedling after 4 days of treatments and 4 days of recovery. Irradiance levels were set at 0, 20, 50, 100, 200, 300, 500, 800, 1000, 1500 and 2000 μmol photon m^−2^s^−1^ for light response curve measurement. CO_2_ concentration was set at 400 μmol mol^−1^ with air temperature and relative humidity set at the greenhouse conditions. Leaves were allowed to acclimate to each irradiance level for about 2 min before reading and irradiance was increased successively from 0 to 2000 μmolm^−2^s^−1^.

CO_2_ concentrations were set at 0, 50, 100, 200, 400, 600, 800, 1000, 1200, 1500 and 2000 μmol mol^−1^ and irradiance levels were set at 1000 μmol m^−2^s^−1^ for CO_2_ response curve measurements. Leaves were allowed to acclimate to each CO_2_ level for about 2 min before reading. The first CO_2_ concentration was set at 400 μmol mol^−1^ which approached the greenhouse CO_2_ concentration and increased to 2000 μmol mol^−1^ and then successively decreased from 2000 to 0 μmol mol^−1^.

These light response curves and CO_2_ response curves were measured on each of the two leaves and replicated three times.

### Measurements of chlorophyll content and carotenoid content

Leaf tissues (0.5 g) supernatant was extracted with 80% v/v acetone and absorbance was measured with a UV–visible spectrophotometer at 663, 645, and 470 nm. Total chlorophyll and carotenoid contents were calculated following the procedure described by Lichtenthaler and Wellburn^44^.

### Measurements of O_2_•^**−**^ producing rate in leaf extracts and MDA conten**t**

The O_2_•^−^ production rate was measured by analyzing nitrite formation from hydroxylamine in the presence of O_2_•^−^ 45. Each 0.5 g of frozen leaf segment was homogenized with 3 mL of 65 mM potassium phosphate buffer (pH 7.8) and centrifuged at 5,000 *g* for 10 min. The incubation mixture contained 0.9 mL of 65 mM phosphate buffer (pH7.8), 0.1 mL of 10 mM hydroxylamine hydrochloride, and 1 mL of the supernatant. After incubation at 25 °C for 20 min, 17 mM sulfanilamide and 7 mM R-naphthylamine were added to the incubation mixture. Ethylether in the same volume was added and centrifuged at 1,500 *g* for 5 min. The absorbance in the aqueous solution was read at 530 nm.

MDA content was determined according to Hodges *et al*.^46^. Leaf samples of 0.3 g were ground with 3 mL ice-cold 25 mM HEPES buffer (pH 7.8) containing 0.2 mM EDTA and 2% PVP. The homogenates were centrifuged at 4 °C for 20 min at 12,000 *g*, and the resulting supernatants were used for MDA analysis. Samples were mixed with 10% TCA containing 0.65% 2-thiobarbituric acid (TBA) and heated at 95 °C for 25 min. MDA content was calculated by correcting for compounds, other than MDA, that absorb at 532 and 600 nm, by subtracting the absorbance at 532 and 600 nm of a solution containing plant extract incubated without TBA from an identical solution containing TBA.

### Measurements of proline and soluble protein

To determine the free proline level, 0.5 g of leaf sample from each group was homogenized in 3% (w/v) 5-sulfosalicylic acid, after which the homogenate was filtered through filter paper^47^. The mixture was heated at 100 °C for 1 h in a water bath after the addition of ninhydrin acid and glacial acetic acid. The reaction was then stopped in an ice bath. The mixture was extracted with toluene, and the absorbance of the fraction with toluene aspired from the liquid phase was read at 520 nm. The proline concentration was determined using a calibration curve^48^.

For biochemical assays, each 0.5 g sample of leaf material was homogenized in 3 mL 25 mM HEPES buffer (pH 7.8) containing 0.2 mM EDTA and 2% (w/v) polyvinylpyrrolidon. The homogenate was centrifuged for 20 min at 12,000 *g* and the supernatant obtained was used for enzyme analysis. All operations were performed at 0–4 °C. An aliquot of the extract was used to determine protein content according to Bradford^49^, using bovine serum albumin as the standard.

### Glutathione assays

For the measurement of GSH and GSSG, plant leaf tissue (0.2 g) was homogenized in 2 mL of 2% metaphosphoric acid containing 2 mM EDTA and centrifuged at 4 °C for 10 min at 14,000 *g*. After neutralization with 0.5 M phosphate buffer (7.5), 0.1 mL of the supernatant was added to a reaction mixture containing 0.2 mM NADPH, 100 mM phosphate buffer (pH 7.5), 5 mM EDTA, and 0.6 mM 5,5′dithio-bis (2-nitrobenzoic acid). The reaction was started by adding 3 U of GR and was monitored by measuring the change in absorbance at 412 nm for 1 min. For the GSSG assay, GSH was masked by adding 20 μL of 2-vinylpyridine to the neutralized supernatant, whereas 20 μL of water was added for the total GSH assay. GSH level was obtained by subtracting the GSSG levels from the total level^50^.

### Antioxidant enzyme activity assay

For the enzyme assays, 0.3 g of leaf sample was ground in 3 mL of ice-cold 25 mM HEPES buffer (pH 7.8) containing 0.2 mM EDTA, 2 mM AsA, and 2% PVP. The homogenates were centrifuged at 4 °C for 20 min at 12,000 *g*, and the supernatants were used for the determination of enzymatic activity. SOD activity was measured in a reaction mixture containing 50 mM phosphate buffer (pH 7.8), 0.1 mM EDTA, 13 mM methionine, 75 μM nitroblue tetrazolium (NBT), 2 μM riboflavin, and 50 μl enzyme aliquot^51^. One unit of SOD activity was defined as the amount of enzyme required to cause a 50% inhibition of the rate of p-nitro blue tetrazolium chloride reduction at 560 nm. The method of Cakmak & Marschner^52^, with some modifications, was used to determine the activity of G-POD. The reaction mixture contained 25 mM phosphate buffer (pH 7.0), 0.05% guaiacol, 1.0 mM H_2_O_2_ and 100 μl enzyme extract. The increase in absorbance at 470 nm caused by guaiacol oxidation (E = 26.6 mM cm^−1^) was used to determine the G-POD activity. CAT was assayed as described by Durner & Klessing^53^, and the activity was determined as a decrease in the absorbance at 240 nm for 1 min following the decomposition of H_2_O_2_. APX was measured according to Nakano & Asada^54^ by monitoring the rate of ascorbate oxidation at 290 nm. GR activity was measured according to Halliwell & Foyer^55^ based on the rate of decrease in the absorbance of NADPH at 340 nm.

### RNA extraction and RT-PCR for gene expression analysis

To determine the effect of HT stress on the transcription levels of Calvin cycle enzyme genes and antioxidant enzyme genes and their changes after GSH application, we tested photosynthetic genes including those encoding ribulose-1,5-bisphosphate carboxylase/oxygenase activase (*RCA*), *RBCL and RBCS* involved in CO_2_ fixation. Other tested genes encode fructose-1,6-bisphosphate aldolase (*FBPaldolase*), which catalyze the conversion of two triose-3-phosphates into FBP. *FBPase* and *SBPase* catalyze the hydrolysis of FBP and SBP to Fru6P and Sed7P, respectively. Meanwhile, the tested antioxidant enzymes genes included *Cu/Zn*-*SOD*, *G-POD*, *CAT*, *cAPX* and *GR*. Total RNA was extracted using an RNA extraction kit (Axgen, Union City, CA) according to the supplier’s instructions. Contaminated DNA was removed with a purifying column. One microgram of total RNA was reverse-transcribed with a ReverTra Ace qPCR RT Kit (Toyobo, Japan) following the supplier’s recommendations. The gene-specific primers used for the amplification were determined on the basis of gene or EST sequences and are listed in Table S1, Supporting Information.

Quantitative real-time polymerase chain reaction (PCR) was performed with an iCycler iQ 96-well real-time PCR Detection System (Bio-Rad, Hercules, CA). PCR products were amplified using the SYBR Green PCR Master Mix (Applied Biosystems, Foster City, CA) in 25 μl of qRT-PCRs. The PCR conditions consisted of denaturation at 95 °C for 3 min, followed by 40 cycles of denaturation at 95 °C for 30 s, annealing at 58 °C for 30 s and extension at 72 °C for 30 s. To minimize sample variations, the mRNA expression of the target gene was normalized relative to the expression of the actin housekeeping gene. A quantification of mRNA levels was performed according to the method of Livak & Schmittgen^56^.

### Statistical analysis

Analysis of variance (ANOVA) was conducted using the Statistical Analysis System (SAS version 9.3) (SAS Institute Inc., Cary, NC). Each value was presented as the mean ± standard deviation (SD), with a minimum of three replicates. Differences between treatment means were tested by the Least Significant Difference (LSD) method at α = 0.05 level of significance. The data were plotted using Origin 7.0 software (Origin Lab, Northampton, MA, USA).

Relationships between the leaf photosynthetic rate (*P*_*n*_) and irradiance under four treatments were analyzed with a rectangular hyperbolic equation^29^:


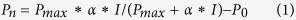


where *P*_*max*_ is the maximum leaf photosynthetic rate, *α* is quantum yield, *I* is irradiance, and *P*_*0*_ is the leaf photosynthetic rate when *I* = 0.

Relationships between *P*_*n*_ and intercellular CO_2_ concentration (*Ci*) of cucumber leaves were also analyzed by CO_2_ response curve. The maximum velocity of RuBP carboxylation by Rubisco (*V*_cmax_) and the maximum potential rate of electron transport contributing to RuBP regeneration (*J*_max_) were estimated by non-linear regression techniques, based on the equations of Harley *et al*.^57^ and Manter & Kerrigan^58^.

## Additional Information

**How to cite this article**: Ding, X. *et al*. Exogenous glutathione improves high root-zone temperature tolerance by modulating photosynthesis, antioxidant and osmolytes systems in cucumber seedlings. *Sci. Rep.* **6**, 35424; doi: 10.1038/srep35424 (2016).

## Supplementary Material

Supplementary Information

## Figures and Tables

**Table 1:** Effects of glutathione (GSH) application on plant growth of cucumber 4 days after the high root-zone temperature treatment, and 4 days recovery.

Treatment	Plant Height (cm)	Stem Diameter (cm)	Shoot Fresh Weight (g)	Shoot Dry Weight (g)	Shoot Water Content (%)
4 days after the high root zone temperature treatment					
NT	60.7 ± 3.2a	0.70 ± 0.03a	88.7 ± 5.3a	8.6 ± 0.2a	90.3 ± 0.4b
NT+GSH	64.3 ± 2.5a	0.71 ± 0.02a	89.2 ± 3.9a	8.7 ± 0.3a	90.4 ± 0.3b
HT	51.7 ± 2.5b	0.64 ± 0.02b	54.9 ± 3.1b	5.8 ± 0.4c	89.4 ± 0.1c
HT+GSH	63.7 ± 1.5a	0.69 ± 0.01a	86.1 ± 5.5a	7.0 ± 0.4b	91.9 ± 0.1a
4 days after recovery					
NT	91.7 ± 1.2a	0.74 ± 0.02a	150.1 ± 6.1a	15.4 ± 1.2a	89.8 ± 1.1a
NT+GSH	93.0 ± 3.0a	0.75 ± 0.02a	151.0 ± 3.1a	15.5 ± 1.1a	89.7 ± 1.2a
HT	69.7 ± 1.5b	0.65 ± 0.01c	99.9 ± 2.8c	9.6 ± 0.4c	90.4 ± 0.4a
HT+GSH	90.0 ± 2.0a	0.70 ± 0.03b	131.9 ± 7.2b	12.1 ± 1.6b	90.9 ± 1.7a

The experiments consisted of four treatments: NT: normal temperature; NT+GSH: normal temperature plus exogenous GSH treatment; HT: high root-zone temperature treatment; and HT+GSH: high root-zone temperature plus exogenous GSH treatment. Data represent the mean ± SD (n = 3). Different letters indicate significant differences at P < 0.05 based on the Least Significant Difference test.

**Table 2:** Effects of GSH application on *P*_*0*_ (the leaf photosynthetic rate when irradiance = 0), *P*_*max*_ (the maximum leaf photosynthetic rate), *α* (quantum yield), *V*_max_ (the maximum velocity of RuBP carboxylation by Rubisco) and *J*_max_ (the maximum potential rate of electron transport contributing to RuBP regeneration) of cucumber 4 days after the high root-zone temperature treatment, and 4 days recovery.

Treatment	*P*_*0*_ (μmol CO_2_ m^−2^s^−1^)	*α*	*P*_*max*_ (μmol CO_2_ m^−2^s^−1^)	*V*_*c,max*_ (μmol m^−2^s^−1^)	*J*_*max*_ (μmol m^−2^s^−1^)
4 days after the high root zone temperature treatment					
NT	2.415 ± 0.199a	0.0794 ± 0.0035a	26.61 ± 0.34a	47.30 ± 1.32a	126.63 ± 12.74a
NT+GSH	2.158 ± 0.193a	0.0775 ± 0.0033a	27.17 ± 0.34a	47.54 ± 1.58a	136.53 ± 6.01a
HT	1.033 ± 0.090b	0.0194 ± 0.0013c	8.58 ± 0.19c	20.42 ± 4.58c	45.51 ± 4.88c
HT+GSH	2.174 ± 0.094a	0.0556 ± 0.0016b	19.20 ± 0.16b	40.73 ± 1.50b	97.81 ± 6.68b
4 days after recovery					
NT	2.241 ± 0.054a	0.0769 ± 0.0009a	29.20 ± 0.10a	46.43 ± 2.92a	131.63 ± 6.86a
NT+GSH	1.942 ± 0.229a	0.0802 ± 0.0038a	29.16 ± 0.41ab	46.27 ± 2.55a	128.64 ± 7.80a
HT	2.285 ± 0.169a	0.0721 ± 0.0029a	24.78 ± 0.29c	40.24 ± 0.89b	101.35 ± 7.44c
HT+GSH	2.063 ± 0.213a	0.0723 ± 0.0034a	27.77 ± 0.40b	41.33 ± 3.20b	110.86 ± 12.68b

Data represent the mean ± SD (n = 3). Different letters indicate significant differences at P < 0.05 based on the Least Significant Difference test.

**Table 3:** Effects of GSH application on chlorophyll content, carotenoid content, soluble protein content, and proline content of cucumber 4 days after the high root-zone temperature treatment.

Treatment	Chlorophyll Content (mg·g^−1^ FW)	Carotenoid Content (mg·g^−1^ FW)	Soluble Protein Content (mg·g^−1^ FW)	Proline Content (μg·g^−1^ FW)
NT	3.14 ± 0.03c	0.47 ± 0.006b	17.35 ± 0.56c	33.77 ± 0.93c
NT+GSH	3.52 ± 0.04a	0.50 ± 0.007a	17.52 ± 1.20c	34.50 ± 0.71c
HT	2.96 ± 0.02d	0.45 ± 0.002c	20.02 ± 1.27b	41.85 ± 1.77b
HT+GSH	3.46 ± 0.02b	0.47 ± 0.002b	24.15 ± 0.49a	63.54 ± 6.84a

Data represent the mean ± SD (n = 3). Different letters indicate significant differences at P < 0.05 based on the Least Significant Difference test.
