# Supplementary material for: Exogenous glutathione improves high root-zone temperature tolerance by modulating photosynthesis, antioxidant and osmolytes systems in cucumber seedlings
Source: Sci Rep. 2016 Oct 18;6:35424. doi: 10.1038/srep35424 (PMC5067582; doi:10.1038/srep35424)
Supplement: Supplementary Information [file srep35424-s1.pdf]

Exogenous glutathione improves high root-zone temperature tolerance by modulating photosynthesis and antioxidant and osmolytes systems in cucumber seedlings

Xiaotao Ding, Yuping Jiang, Lizhong He, Qiang Zhou, Jizhu Yu, Dafeng Hui and Danfeng Huang

Supplemental Table S1. Primers used for real time RT-PCR assays

Gene	Forward primer	Reverse primer
actin	5’-TGGACTCTGGTGATGGTGTTA-3’	5’-CAATGAGGGATGGCTGGAAAA-3’
*rbc*L	5’-ACCGATGGGCTTACCAGTCT-3’	5’-ATTCGCAAATCCTCCAGACG-3’
*rbc*S	5’-ATGGGTTCCCTGCGTTGA-3’	5’-CCTGAGATGAGTCGGTGC-3’
rca	5’-GCTGACAACCCAACCAA-3’	5’-CATCCGACCATCACGAA-3’
SBPase	5’- CAGGGTTATCAAATGTGG-3’	5’- GGAGTGAAGGGAAGCGAC-3’
FBP-aldolase	5’-CTCGTGCTGCTGCTTACT-3’	5’-CTGCCCAAACTTTCTGTG-3’
FBPase	5’-GGGAGAGGACCAGAAAAA-3’	5’-GGCTGTAGATGCCAAAGA-3’
POD	5’-AGTGCTTGTCCAGGAGTTGA-3’	5’- AGGGATGAAGTGGGATAAAG -3’
CAT	5’-ATGCTGGAAGAGGAGGCTAT-3’	5’- ATGGTGAGGACATTTGGGAG -3’
cAPX	5’- ATGGGAAAGTGCTACCCTGTT-3’	5’- ACAATGTCCTGGTCCGAAAG -3’
Cu/Zn- SOD	5’-CAAGTTAACGCATGGTGCTC-3’	5’-GGCAGTTATGTTTCCCAGGT-3’
GR	5’- GAGTTTCCTAAGCGTGTT-3’	5’-TTTGTTCTTGGGTGCA-3’
